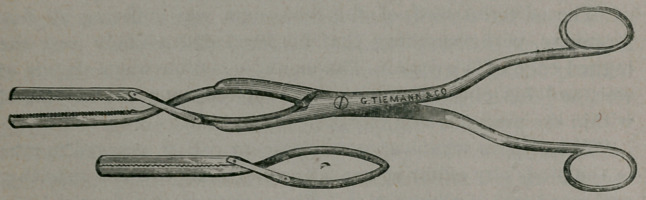# Dr. McLaughlin’s New Hemostatic Forceps

**Published:** 1890-01

**Authors:** J. W. McLaughlin

**Affiliations:** Austin


### A NEW SURGICAL INSTRUMENT.

[Devised by Dr. J. W. McLaughlin, Austin, and manufactured by George Tiemann & Co.]

The accompanying cut represents a clamp forcep partly open, in the grasp of its handle, and another clamp forcep closed and separate from the handle. Each set contains three pairs, or six clamps, and one detachable handle.

The length of the largest clamp is 4½ inches; the blades of the clamp are two inches in length.

The largest clamps are designed particularly for clamping the broad ligiments in vaginal hysterectomies.

Any of the clamps may be used to control hemorrhage in laparotomies or in clamping an adherent omentum, when it is necessary to cut this and quickly reach the pedicle of an ovarian or other abdominal tumor.

The advantages claimed for these, over other forceps in clamping the broad ligaments, are ease of their application; fewer instruments required and a better vaginal toilet secured.

If the vessels of the parametrium are secured by a ligature on each side, passed from the lateral fornices of the vagina; and the uterus separated from its parametrical, vaginal and vesical attachments, the blades of the largest clamp can be easily placed over the broad ligament. One blade should pass in front, the other behind the broad ligament.

When the clamps are thus applied, the uterus can be separated from its remaining attachments without the least danger from hemorrhage.

The two clamp forceps, one on either side, will rest entirely within the cavity and the vagina; they will afford good drainage and not interfere with an antiseptic dressing being applied.

In an operation for the removal of a hemorrhoidal tumor recently performed by Dr. McLaughlin, after thoroughly stretching the sphincter muscle, the tumor was drawn down and the smallest clamp applied to its base; the tumor was then cut away, the edges of the wound stitched with cat gut; one or two ligatures were required, passed back of the clamp to prevent hemorrhage, the clamp removed and ligature tied.

In this way the operation can be made aseptic. To those who clamp the foreskin before excising it, in circumcision, this instrument will recommend itself. The instruments can be had of Tieman & Co., of New York. They predict a big sale for it.

## Figures and Tables

**Figure f1:**